# A Treatise on Cholera

**Published:** 1885-10

**Authors:** 


A Treatise on Cholera. Its origin, history, lesions, prevention, and treatment, by ALFRED STILLE, M. D., L. L. D. Professor Emeritus of the Theory and Practice of Medicine, in the University of Pennsylvania. First edition. Philadelphia: Lee Brothers & Co. 1885.

This volume will be of great service to the profession, as it is very comprehensive. No library at present is complete without such a book, and this seems to us to be one of the very best on the subject. The author will do much good if he accomplishes what he hopes to, "to disabuse the medical profession of the erroneous notion that the disease even originates de novo. To maintain the necessity of quarantine, not in the literal but in the official sense of that word." The chapters on this subject are very logical, and cannot fail to convince any one of their truth and importance, if studied carefully. The chapters on prevention and treatment are admirable. We can certainly recommend this to the profession as up to the standard of the other published writings of this celebrated author.

